# Immediate Psychological Responses, Stress Factors, and Coping Behaviors in Military Health-Care Professionals During the COVID-19 Pandemic in Tunisia

**DOI:** 10.3389/fpsyt.2021.622830

**Published:** 2021-05-20

**Authors:** Hela Slama, Hamdi El Kefi, Karima Taamallah, Nejla Stambouli, Anis Baffoun, Walid Samoud, Chaker Bechikh, Abdelaziz Oumaya, Khaled Lamine, Mohamed Jalel Hmida, Hichem Slama, Mustapha Ferjani, Hedi Gharsallah

**Affiliations:** ^1^Psychiatric Department, Military Hospital of Tunis, Tunis, Tunisia; ^2^Research Unit UR17DN05 Medical Support to the Armed Forces in Operations and Disaster Situations, Military Hospital of Tunis, Tunis, Tunisia; ^3^Faculté de Médecine de Monastir, Université de Monastir, Tunis, Tunisia; ^4^Faculté de Médecine de Tunis, Université de Tunis El Manar, Tunis, Tunisia; ^5^Cardiology Department, Military Hospital of Tunis, Tunis, Tunisia; ^6^Department of Anaesthesiology and Intensive Care, Military Hospital of Tunis, Tunis, Tunisia; ^7^Emergency Department, Military Hospital of Tunis, Tunis, Tunisia; ^8^Department of Neuropsychology and Speech Therapy, Erasme Hospital, Université Libre de Bruxelles, Brussels, Belgium

**Keywords:** COVID-19, coping, emotion, healthcare workers, stress

## Abstract

**Objective:** The COVID-19 epidemic began in Tunisia in March 2020; health-care workers (HCWs) were suddenly confronted with a particularly stressful situation. The aim of this study was to assess the psychological responses of HCWs during the epidemic, determine the stressors and identify ways to cope.

**Methods:** This cross-sectional study used an online questionnaire that included 62 questions. ANOVAs and *t*-tests were used to compare the responses between professional groups, age groups, and genders.

**Results:** Questionnaires were completed by 368 HCWs. HCWs believed they had a social and professional obligation to continue working long hours (95.3%). They were anxious regarding their safety (93.7%) and the safety of their families (97.8%). Youthful age (*p* = 0.044) and female gender (*p*s <0.046) were identified as stressors. The availability of personal protective equipment (PPE; 99.7%) and good communication between colleagues (98.1%) and managers (91.6%) were important protective factors. Family and friend support (95.9%), following strict protective measures (99.4%), knowing more about COVID-19 (94.8%), adopting a positive attitude (89.6%), and engaging in leisure activities (96.1%) helped in dealing with this epidemic.

**Conclusion:** This study highlights the importance of providing HCWs with infection control guidelines and adequate PPE. Communication and support within the team and maintaining family support help in coping with this stressful situation.

## Introduction

In November 2019, a new coronavirus disease (COVID-19) was reported for the first time and then became widespread in Wuhan, China. The disease spread rapidly across China and elsewhere and has created unprecedented challenges to health-care systems. The coronavirus epidemic reached Tunisia in March 2020, with the first case being detected on March 12. Given the magnitude of the global impact of this pandemic, total containment was declared on March 13, 2020 in Tunisia (1).

COVID-19 was an unknown and dangerous disease that our country had not faced before, and the Tunisian national health system was severely put under the test. Health-care workers (HCWs) were suddenly faced with a particularly stressful situation, with a high risk of contamination, insufficient access to protective equipment, physical exhaustion, social isolation, and extreme workload. HCWs have been redeployed to areas outside their usual clinical expertise, often working overtime, sometimes for a full week without returning home. Some HCWs were quickly contaminated, which led to anxiety within the teams. As a result, HCWs have experienced anxiety coexisting with prolonged conditions of uncertainty. Unfortunately, little is known about how best to prepare for and cope with these conditions and how to maximize the HCWs' health and well-being and their long-term psychological resilience. It has become clear to decision-makers that implementing an intervention that corresponds to HCWs' needs and expectations, to allow them to better live through this crisis and to continue to work effectively, has become an urgent matter. Before effective interventions can be developed to support health-care professionals, it is essential to understand the HCWs' specific sources of anxiety and concerns, which may vary from one culture or situation to another. Recognizing sources of stress allows decision-makers to develop appropriate ways to address these concerns and to provide specific support (2, 3). For a low-income country such as Tunisia, the focus was for the first time on the psychological state of health professionals and how to improve it.

### Aim

The aim of this study is to identify the psychological responses, stress factors and coping strategies during the COVID-19 epidemic, in military health-care personnel in Tunisia.

## Methods

### Study Design

We conducted a cross-sectional study over the course of 1 month from May 1 to May 31, 2020. We used an online questionnaire approved by the local ethics committee (local committee for the protection of personnel of the military hospital of TUNIS CLPP). All participants gave their informed consent electronically before answering the questionnaire online.

#### Inclusion/Exclusion Criteria and Procedure

Participants of the study were made up of active health-care staff [doctors, residents, nurses, medical technicians and administrative staff working in the medical departments (medical secretaries)], including frontline and non-frontline staff working at the military hospital in Tunis and in seven other military care structures throughout Tunisia (Table 1). Participants with incomplete responses to the questionnaire were excluded.

**Table 1:** Medical staff demographics (*n* = 368).

**Characteristic**	**Value**
Mean age (years) ± SD	36.34 ± 11.14
**Gender**, ***N*** **(%)**	
Female	145 (39.4%)
Male	223 (60.6%)
Married, *N* (%)	211 (57.3%)
Having children, *N* (%)	203 (57.1%)
**Life habit**, ***N*** **(%)**	
Tobacco	69 (18.8%)
Alcohol	16 (4.3%)
Tobacco and alcohol	19 (5.2%)
Nothing	264 (71.7%)
**Professional**, ***N*** **(%)**	
Nurse	150 (40.8%)
Doctor	138 (37.5%)
Medical resident	34 (9.2%)
Medical technician	26 (7.1%)
Hospital staff	20 (5.4%)
**Workspace**, ***N*** **(%)**	
Military Hospital of Tunis	279 (75.8%)
Medical Centers of the Military Units	48 (13%)
Military Clinic	21 (5.7%)
Specialized Military Medical Center	12 (3.3%)
Military Hospital of Bizerte	6 (1.6%)
Military Hospital of Gabes	2 (0.5%)
**Department**, ***N*** **(%)**	
Medicine department	158 (42.9%)
Surgery department	58 (15.8%)
Intensive care unit	51 (13.9%)
Emergency department	29 (7.9%)
Laboratory	14 (3.8%)
Administration	13 (3.5%)
Others	45 (12.2%)
Work experience (years), mean (SD)	11.61 (9.92)
**Work in COVID unit**, ***N*** **(%)**	
Yes	109 (29.6%)
No	259 (70.4%)
**Manage patient with suspected or confirmed coronavirus**, ***N*** **(%)**	
Yes	168 (45.7%)
No	200 (54.3%)
**Means of conveyance**, ***N*** **(%)**	
Car	225 (61.1%)
Military bus	64 (17.4%)
Mass transit	62 (16.8%)
On foot	11 (3%)
Bike or motorcycle	6 (1.6%)

*COVID-19, Coronavirus disease 2019; SD, Standard deviation*.

At the onset of the epidemic, all clinical staff were asked to work periodically in the COVID units. Therefore, we did not separate those who worked on the frontline from those who had not yet worked. At the time of the study, 109 health-care workers out of 368 had already worked in the COVID units (see Table 1, Work in COVID unit).

The questionnaire was emailed to health-care workers of the military health-care structures in Tunisia. For those who did not have an e-mail address, a phone message was sent with the link to the questionnaire.

### Study Survey

The study survey included two parts:

- First part: Questions assessing socio-demographic data (e.g., age, sex, profession, education level, marital status)

- Second part: Questions assessing psychological responses, stress factors and coping strategies

As there were no COVID-19-specific, validated questionnaires in Arabic assessing psychological responses, stress factors and coping strategies, we developed an instrument based on consensus from a multidisciplinary team. The multidisciplinary team consisted of four psychiatrists, a neuropsychiatrist, three resuscitators, a researcher in biomedical sciences, and a medical director of care delivery from the hospital. A medical doctor in charge of the COVID unit conducted this study. The questionnaire was based on previous literature, knowledge of the field, linguistic and cultural aspects. The authors adapted a questionnaire previously developed by Lee et al. (2), which was used to assess medical staff during the 2003 Severe Acute Respiratory Syndrome (SARS) epidemic and consisted of 72 questions. The same questionnaire was modified to a 67-item questionnaire and was used by Haozheng Cai et al. (3) during the COVID-19 pandemic in March 2020 and was also used to develop this questionnaire. In a first step, the multidisciplinary team organized a debriefing with members of the medical staff. Topics related to their experience with this epidemic were discussed, such as their psychological experience, stress factors, coping strategies and possible prevention or intervention measures for staff. From this debriefing, 62 questions were selected, taking into account Tunisian cultural specificity and experience in the field at this time of the pandemic.

The questionnaire was comprised of four sections that explored immediate reactions to the mission of health-care workers during the COVID-19 outbreak, stressors, protective factors, coping behaviors and wellness resources. Each question had a four-point Likert scale [**0** = not at all; **1** = slightly (equivalent to mild); **2** = moderately; **3** = very much]. The percentage of participants for each item was calculated from the sum of positive responses (more than 0). The intensity or degree of importance of the item was calculated by averaging all the scores. The higher the mean, the more intense the item.

#### Immediate Reactions to the Mission of Health-Care Workers During the COVID-19 Outbreak

The first section of the questionnaire consisted of 14 questions (Q1–Q14; Table 2). Ten questions examined cognitive, emotional reactions and avoiding behaviors of the HCWs during their work. Four questions explored their experiences and feelings pertaining to their families during this period, inspired by the Parental Burnout Assessment (PBA) (4). Indeed, during the debriefing with the multidisciplinary team, we noticed a major concern of the caregivers regarding their paternal function and their feeling of exhaustion.

**Table 2:** Immediate reactions to the mission of health-care workers during COVID-19 outbreak among professional groups.

	Mean (SD)^*^					***P*-value**	Total mean (SD)^*^	Frequency^**^ (%)
	**Nurses *N* = 150**	**Doctors** ***N* = 138**	**Residents *N* = 34**	**Health technicians** ***N* = 26**	**Administrators *N* = 20**			
Q1-You think that your current front-line job comes from your social and moral responsibility	2.1 (0.83)	2.25 (0.91)	1.52 (0.82)	2.13 (0.67)	2 (1.16)	0.001	2.1(0.89)	95.3
Q2-You have felt nervous or frightened in the ward	1.03 (0.77)	0.89 (0.69)	1.35 (0.72)	1.19 (0.89)	1.1 (1.07)	0.02	1.02 (0.77)	76.3
Q3-You were unhappy about working overtime during the outbreak	1.04 (0.8)	0.60 (0.94)	0.85 (0.92)	1 (0.93)	0.9 (0.91)	0.000	0.85 (0.82)	61.4
Q4-You expect recognition or receiving bonus or compensation for your work from the hospital authorities	1.20 (1.07)	0.68 (0.94)	1.05 (0.88)	0.8 (0.93)	1.35 (1.18)	0.000	0.97 (1.03)	58.4
Q5-You try to avoid COVID-19 positive patients or suspects of infection	1.08 (1.12)	0.68 (0.84)	1.11 (1.06)	1.69 (1.22)	1.85 (1.22)	0.000	1.02 (1.07)	58.4
Q6-You try to avoid your colleague working in COVID unit	0.8 (0.9)	0.53 (0.88)	0.73 (0.99)	1 (0.89)	1.4 (1.23)	0.001	0.75 (0.97)	45.9
Q7-You tried to avoid working in COVID unit	0.37 (21.7)	0.31 (0.70)	0.96 (1.08)	0.6 (1.09)	0.8 (1.19)	0.000	0.43 (0.85)	26.5
Q8-You want to stop your present job to avoid being at hospital	0.12 (0.38)	0.02 (0.14	0.14 (0.43)	0.15 (0.36)	0.8 (1.19)	0.000	0.12 (0.44)	9.5
Q9-You've been thinking about stopping your job if the epidemic suddenly gets worse	0.16 (0.49)	0.07 (0.26)	1.7 (0.45)	0.19 (0.36)	0.7 (1.03)	0.000	0.16 (0.48)	12.2
Q10-You feel angry because your workload is greater and more dangerous than other doctors who have not been exposed to COVID-19	0.8 (0.84)	0.56 (0.79)	0.79 (0.76)	0.57 (0.49)	0.75 (1.03)	0.127	0.69 (0.48)	50
Q11-When I come home, I feel unable to take care of my children or family members and have no energy	1.1 (1.03)	0.98 (0.88)	1.2 (1.03	1.26 (1.04)	1.25 (0.85)	0.423	1.10 (0.97)	68.7
Q12-I'm happy when I'm with my children and family members	1.4 (1.14)	2.17 (0.95)	1.73 (1.02	1.92 (1.09)	2.3 (0.92)	0.000	1.8 (0.97)	83.6
Q13-I no longer think I'm as good a mother/father as I have been to my kids	0.8 (0.88)	0.65 (0.86)	0.68 (0.87)	0.25 (0.44)	1.25 (0.95)	0.037	0.71 (1.09)	49.7
Q14-I feel that I take care of my children in a mechanical way and that I'm not able to show them my affection	1.07 (0.96)	0.78 (0.58)	0.82 (0.79)	0.8 (1.02)	0.95 (0.88)	0.08	0.91 (0.91)	59.7

*SD, standard deviation*.

* *Mean of total score of the item [rated on a four-point scale (0 = not at all; 1 = slightly; 2 = moderately; 3 = very much)]*.

** *Frequency represents the number of subjects who responded more than 0 to the specific item*.

#### Stressors

The second section examined 18 possible factors that could induce stress.

#### Protective Factors

The third section consisted of 16 questions aimed at identifying factors that could reduce stress.

#### Coping Behaviors Used by Health-Care Workers as a Resource for Well-Being

The fourth section consisted of 14 questions designed to identify coping behaviors in response to stress.

### Statistical Analysis

Statistical analysis of the data was performed using SPSS 22.0 software. Descriptive statistics were used to present the data collected during the survey, all continuous variables were expressed as mean ± SD, and categorical variables as percentages. To compare responses across professional groups, age groups, and gender for the four sections of the second part of the questionnaire, mean comparisons were performed for each item. Student's *t*-tests, one-way analyses of variance (ANOVA) and Tukey's *post-hoc* tests for the most significant differences were used to analyze the data.

## Results

### Demographic Characteristics

The demographic characteristics are shown in Table 1.

A total of 368 questionnaires were completed out of a total of 544 sent (67.6%). The mean age of the participants was 36.34 ± 11.14 years old (20–68 years old) with 60.6% being male. There was no difference in age between male and female participants. Supplementary Table 1 shows demographic characteristics by gender. The percentage of married participants was 57.3% (*n* = 211) and 57.1% had at least one child (*n* = 203). Participants from the Military Hospital of Tunis were 75.8% (*n* = 275), while participants from the other medical centers of the military units represented 24.2% (*n* = 89). Professional work experience ranged from 0 to 40 years (11.6 ± 9.92 years).

Approximately half of the HCWs (52.2%) worked in COVID units or treated a confirmed or suspected case of COVID-19. Supplementary Table 2 shows demographic characteristics in frontline and non-frontline workers.

During the study, all participants resided primarily in their homes. Travel to the hospital was done by personal means (car, motorcycle, bicycle, on foot) for 65.7% of the participants. Mass transit or military bus was used by the remaining 34.3%.

### Immediate Reactions to the Mission of Health-Care Workers During the COVID-19 Outbreak

Table 2 indicates means, standard deviations, and frequency for the questions assessing the immediate reactions of health-care workers. Detailed means among professional groups are also presented in Table 2. Our results showed that 76.3% of participants felt nervous or frightened in the ward (Q2). There was a difference among the various health-care professions [*F*_(4, 363)_ = 2.96; *p* = 0.02]. *Post-hoc* comparisons indicated that residents were more frightened than nurses (*p* = 0.016).

Responses were influenced by age [*F*_(3, 364)_ = 2.75; *p* = 0.04], gender [*t*_(366)_ = 4.435, *p* < 0.001] but not by marital status (*p* = 0.168). *Post-hoc* comparisons indicated that HCWs over 50 years felt less nervous or frightened than those between 20 and 30 years old (0.77 ± 0.89 vs. 1.12 ± 0.79; *p* = 0.033). Women felt more nervous or frightened at work (1.24 ± 0.78 vs. 0.88 ± 0.74). Women were also more dissatisfied with working overtime [Q3; *t*_(366)_ = 2.642, *p* = 0.009; 0.99 ± 0.87 vs. 0.76 ± 0.78], and had a stronger desire to avoid working in the intensive care unit [Q7; *t*_(366)_ = 2.586, *p* = 0.01; 0.61 ± 0.91 vs. 0.35 ± 0.79].

The amount of avoidance behaviors was different among the professions [*F*_(4, 300)_ = 5.246; *p* < 0.001]. *Post-hoc* comparison indicated that residents tried more to avoid working in the COVID units (Q7) compared to doctors (*p* = 0.001) and nurses (*p* = 0.006). Administrators showed a stronger desire to stop working in the hospital [Q8; *F*_(4, 363)_ = 2.655; *p* < 0.001] compared to all other members of the medical staff (nurses, residents, doctors, health technicians; all *p*s < 0.001).

Compared with married staff, single individuals were less happy about working overtime during the outbreak [Q3; *t*_(366)_ = 2.447, *p* = 0.015; 0.97 ± 0.81 vs. 0.76 ± 0.82]. They adopted more avoidance behaviors such as avoiding suspect patients [Q5; *t*_(366)_ = 2.227, *p* = 0.027; 1.17 ± 1.09 vs. 0.91 ± 1.05] or avoiding colleagues working in the COVID units [Q6; *t*_(366)_ = 2.052, *p* = 0.041; 0.88 ± 1.02 vs. 0.67 ± 0.93].

Social and moral responsibility was present in 95.3% of healthcare workers (Q1). A significant difference was found between the different professional groups [*F*_(4, 363)_ = 5.055, *p* = 0.001]. Residents had the lowest social and moral responsibility among care givers with lower scores than nurses, doctors, and health technicians (all *p*s < 0.006) but not than administrators (*p* = 0.763).

While 58% of HCWs expected to receive recognition, bonuses or compensation for their work from hospital authorities (Q4), a significant difference was noted between the different professions [*F*_(4, 363)_ = 5.732, *p* < 0.001]. Nurses and administrators had more concerns regarding extra financial compensation during or after the outbreak compared to doctors (*p* < 0.001 and *p* = 0.49, respectively).

Regarding the four questions exploring the parents' experiences and their feelings, 83.6% of HCWs appear to be happy when they are in the presence of their family and children (Q12), but with significant differences between professional groups [*F*_(4, 363)_ = 8.781, *p* < 0.001]. Nurses were less happy than doctors and administrators (*p* < 0.001 and *p* = 0.011, respectively). More than half of the participants (59,6%) felt as though their caring for their children was mechanical without being able to show their love for them (Q14). In comparison with fathers, mothers had a stronger feeling of not being as good a parent as they were before the epidemic [Q13; *t*_(235)_ = 2.165, *p* = 0.031]. Participant felt that they could no longer care for their children and family and that they had no energy left (Q11; 68.7%). Women expressed this feeling more in comparison with men [*t*_(366)_ = 3.058, *p* = 0.002].

### Stressors

Table 3 presents the stressor questions, mean scores and frequency of responses that were positive (more than 0). Table 2 also presents the mean scores for stressors according to professional groups. Among the main stressors for HCWs we can point to concerns of infecting family members (S2; 97.8%; 2.68 ± 0.72), and colleagues (S1; 94%; 2.14 ± 0.96), concerns of making protection errors that could spread the disease (S4; 96.7%; 2.35 ± 1.39), lack of personal protective equipment (PPE) (S14; 92.1%; 2.24 ± 0.98) and death of their patients (S5; 94.3; 2.24 ± 0.93).

**Table 3:** Stressors during the COVID-19 pandemic among professional groups.

	Mean (SD)^*^					***P*-value**	Total mean^*^ (S.D.)	Frequency^**^ [*N* (%)]
	**Nurse (*N* = 150)**	**Doctor** **(*N* = 138)**	**Resident doctor (*N* = 34)**	**Health technician** **(*N* = 26)**	**Administrative (*N* = 20)**			
S1-Infection of a co-worker	2.17 (1.00)	2.02 (0.95)	2.44 (0.82)	2.26 (0.77)	2.10 (1.07)	0.192	2.14 (0.96)	346 (94.0)
S2-Worried about infecting family	2.69 (0.75)	2.68 (0.66)	2.91 (0.37)	2.65 (0.68)	2.30 (1.17)	0.058	2.68 (0.72)	360 (97.8)
S3-Worried about getting infected	2.08 (0.95)	1.92 (0.90)	1.88 (0.91)	2.00 (1.05)	1.95 (1.23)	0.667	1.99 (0.95)	345 (93.7)
S4-The occurrence of errors at work that can lead to infections	2.34 (0.89)	2.43 (1.94)	2.09 (0.92)	2.34 (0.97)	2.36 (0.83)	0.814	2.35 (1.39)	356 (96.7)
S5-Watching infected patients die	2.17 (1.06)	2.34 (0.80)	2.32 (0.83)	2.06 (0.88)	2.00 (1.00)	0.445	2.24 (0.93)	347 (94.3)
S6-Not knowing when the outbreak will be contained	1.12 (1.10)	1.50 (1.08)	1.67 (0.97)	1.50 (1.14)	1.40 (1.23)	0.014	1.35 (1.10)	259 (70.5)
S7-Participating in the management of infected patients	1.14 (1.31)	1.15 (1.04)	1.58 (0.98)	1.50 (1.14)	1.14 (1.25)	0.219	1.22 (1.17)	238 (64.7)
S8-Lack of specific treatment for COVID	2.08 (0.98)	2.08 (0.92)	1.91 (0.99)	2.50 (0.76)	2.05 (0.94)	0.197	2.09 (0.95)	345 (93.7)
S9-The daily report of the number of new infected cases	2.15 (0.91)	2.18 (1.17)	2.11 (0.80)	2.38 (0.80)	2.25 (0.85)	0.843	2.18 (1.00)	351 (95.4)
S10-Feeling exhausted	1.88 (1.00)	1.65 (1.01)	1.44 (1.02)	1.84 (1.08)	1.75 (1.06)	0.130	1.75 (1.02)	234 (88.0)
S11-Observing symptoms of the disease in your colleagues	1.98 (1.03)	2.00 (0.89)	2.00 (0.98)	2.11 (0.90)	2.20 (1.05)	0.873	2.01 (0.96)	341 (92.7)
S12-Developing symptoms of the disease	2.00 (1.83)	2.10 (0.86)	2.11 (0.84)	2.03 (1.07)	2.25 (1.01)	0.804	2.07 (0.97)	338 (91.8)
S13-Seeing stress or fear from colleagues	1.98 (0.98)	1.89 (0.88)	1.73 (0.96)	2.00 (0.93)	2.00 (0.97)	0.648	1.92 (0.93)	341 (92.7)
S14-Lack of means and protective clothing	2.37 (0.95)	2.26 (0.91)	1.94 (1.07)	2.07 (1.16)	1.95 (1.09)	0.077	2.24 (0.98)	339 (92.1)
S15-Wearing protective clothing for a long time	1.68 (1.07)	1.73 (0.93)	2.26 (0.79)	1.61 (1.13)	1.70 (1.17)	0.040	1.75 (1.01)	317 (86.1)
S16-Working outside of the team and the regular department	1.52 (1.06)	1.39 (1.00)	1.76 (1.01)	1.69 (1.01)	1.25 (1.16)	0.217	1.49 (1.04)	294 (79.9)
S17-Being repeatedly screened for infection	1.32 (1.17)	1.13 (1.00)	1.17 (1.02)	1.11 (1.10)	1.25 (1.20)	0.651	1.21 (1.09)	245 (66.6)
S18-Mandatory sanitary confinement	1.63 (1.13)	1.51 (1.14)	1.58 (1.07)	1.53 (1.13)	1.65 (1.08)	0.923	1.57 (1.12)	284 (77.2)

*S, Stressor; SD, standard deviation*.

* *Mean of total score of the item [rated on a four-point scale (0 = not at all; 1 = slightly; 2 = moderately; 3 = very much)]*.

** *Frequency represents the number of subjects who responded more than 0 to the specific item*.

A difference was noted between the different members of medical staff concerning the uncertainty of when the epidemic would end [S6; *F*_(4, 363)_ = 3.188, *p* = 0.014]. Nurses were significantly less stressed by the uncertainty than doctors (*p* = 0.028). Stress induced by wearing PPE for long periods of time was also significantly influenced by profession [S15; *F*_(4, 363)_ = 2.533, *p* = 0.04]. Residents were less stressed than nurses by PPE (*p* = 0.02).

Women displayed higher mean scores for all stressors. These results were statistically significant for 13 of the 18 stressor questions (Supplementary Table 3).

Staff under 40 years of age were more concerned about infecting family members compared to staff over 50 years of age [S2; *F*_(3, 364)_ = 3.526, *p* = 0.015; *Post-hoc p-*values < 0.044]. We did not find any significant differences between age categories for other stressors.

For a detailed analysis of associations between highest stressors and immediate reactions of health-care workers see Supplementary Table 4.

### Protective Factors

Table 4 presents the protective factor questions, mean scores and frequency of responses that were positive (more than 0). Among protective factors that can reduce the stress of HCWs we can point to good health of co-workers (P3; 97.6%; 2.73 ± 1.78), availability of PPE (P5; 99.7%; 2.67 ± 0.57), recovery of patients (P4; 98.9; 2.60 ± 0.66), decrease in the number of patients infected with SARS-COV2 (P12; 98.9%; 2.58 ± 0.66), support by colleagues and team spirit (P1; 98.1%; 2.50 ± 1.31) and ability to communicate with superiors and commanders (P14, 91.6%; 2.16 ± 0.94).

**Table 4:** Protective factors during the COVID-19 pandemic.

	Mean (SD)^*^	Frequency^**^ [*N* (%)]
P1-Support by colleagues and team spirit	2.50 (1.31)	361 (98.1)
P2-Discussions and jokes between colleagues	2.48 (0.73)	362 (98.4)
P3-The good health of colleagues	2.73 (1.78)	399 (97.6)
P4-Improvement of patient's health and recovery	2.60 (0.66)	364 (98.9)
P5-Availability of protection means	2.67 (0.57)	367 (99.7)
P6-Practice putting on protective gear	2.41 (0.77)	357 (97.0)
P7-Taking preventive treatment	1.84 (1.09)	308 (83.7)
P8 Stress management training	2.09 (0.95)	342 (92.9)
P9-Implementation by the hospital of procedures to combat COVID-19	2.34 (0.82)	355 (96.5)
P10 -Having information about the virus from reliable sources	2.32 (0.86)	351 (95.4)
P11-The good health of your family members	2.01 (0.96)	364 (98.9)
P12-Decrease in the number of infected patients	2.58 (0.66)	364 (98.9)
P13-Obtaining a professional promotion or an incentive bonus	1.67 (1.22)	267 (72.6)
P14-The ability to communicate with superiors and commanders	2.16 (0.94)	337 (91.6)
P15-Confidence in the quality of medical care provided by the hospital to infected health-care personnel	2.25 (0.88)	350 (95.1)
P16-Quality and improved meals in times of pandemic	1.57 (1.12)	316 (85.9)

*P, protective factor*.

* *Mean of total score of the item [rated on a four-point scale (0 = not at all; 1 = slightly; 2 = moderately; 3 = very much)]*.

** *Frequency represents the number of subjects who responded more than 0 to the specific item*.

There were no significant differences between women and men for protective factors (all *p*s > 0.05)

Regarding age categories, obtaining a promotion or award [P13; *F*_(3, 364)_ = 3.522, *p* = 0.015] was less protective against stress for HCWs over 50 years old compared to HCWs under 40 years old (all *p*s < 0.191). Good quality of medical care provided to HCWs in case of COVID-19 contamination [P15; *F*_(3, 364)_ = 4.244, *p* = 0.006] was considered as a more important protective factor by HCWs over 50 years old compared to HCWs under 30 years old (2.55 ± 0.62 vs. 2.09 ± 0.96; *p* = 0.008).

### Coping Behaviors Used by Health-Care Workers as A Resource for Well-Being

Table 5 presents the coping behavior questions, mean scores and frequency of responses that were positive (more than 0). Applying strict protective measures was an important factor in reducing stress (C1; 99.4%; 2.4 ± 0.68), followed by finding information on COVID-19 (C2; 94.8%; 2.06 ± 0.92). Among coping behaviors considered as important by HCWs we can point to performing leisure activities during their free time (C4; 96.2%; 1.95 ± 0.91), discussions with family and friends to relieve stress and obtain support (C5; 95.9%; 1.9 ± 0.88), adopting a positive attitude with self-motivation (C6; 89.6%; 1.62 ± 0.95) and religious practices (C13; 87.2%; 1.54 ± 0.96). Only, 27.17% of HCWs expressed a desire to consult a psychiatrist or a psychologist (C14; 0.35 ± 0.67). Mean scores for coping behaviors including following strict protective measures (C1), performing or participating in leisure activities (C4), discussions with family and friends to relieve stress and obtain support (C5), self-motivation and positive attitude (C6) did not differ between professional groups (all *p*s > 0.05).

**Table 5:** Coping factors during covid 19 pandemic by gender.

	Mean (S.D.)^*^		Total mean^*^ (S.D.)	Frequency^**^ %	***P*-value**
	**Women** **(*N* = 145)**	**Men** **(*N* = 223)**			
C1-Following strict protective measures, such as hand washing, masks, face masks, protective clothing, etc.	2.62 (0.60)	2.4 (0.72)	99.4	2.4 (0.68)	0.002
C2-Learning about COVID-19, its prevention and mechanism of transmission	2.06 (0.87)	2.06 (0.95)	94.8	2.06 (0.92)	0.98
C3-Choosing a more single mode of travel, such as self-driving, and avoid transportation such as subways	2.53 (0.81)	2.35 (0.87)	95.6	2.4 (0.85)	0.055
C4-Doing leisure activities in your free time, such as watching movies, reading, etc.	1.87 (0.88)	2.01 (0.92)	96.1	1.95 (0.91)	0.15
C5-Chatting with family and friends to relieve stress and obtain support	1.9 (0.86)	1.99 (0.89)	95.9	1.9 (0.88)	0.49
C6-Talking to and motivating yourself to face the COVID-19 outbreak with positive attitude	1.65 (0.93)	1.6 (0.97)	89.6	1.62 (0.95)	0.62
C7-Avoiding overtime to reduce exposure to COVID-19 patients in hospital	1.24 (0.87)	1.12 (1.04)	72	1.17 (0.98)	0.21
C8-Avoiding media news about COVID-19 and related fatalities	1.4 (0.90)	1.26 (1.02)	79	1.3 (0.97)	0.19
C9-Venting emotions by crying, screaming etc.	0.76 (0.85)	0.29 (0.65)	33.6	0.47 (0.77)	<0.001
C10-Using means of relaxation and stress management	1.09 (0.90)	0.98 (0.91)	68.7	1.02 (0.90)	0.25
C11-Using treatments or phytotherapy	0.64 (0.89)	0.33 (0.68)	31.2	0.45 (0.97)	<0.001
C12-Drinking alcohol or smoking	0.17 (0.58)	0.51 (0.86)	23.3	0.38 (0.78)	<0.001
C13-Religious practices: prayer, fast,…	1.68 (0.92)	1.45 (0.98)	87.2	1.54 (0.96)	0.02
C14-Seeking help from a psychiatrist or psychologist	0.42 (0.67)	0.30 (0.66)	27.17	0.35 (0.67)	0.12

*C, coping factor*.

* *Mean of total score of the item [rated on a four-point scale (0 = not at all; 1 = slightly; 2 = moderately; 3 = very much)]*.

** *Frequency represents the number of subjects who responded more than 0 to the specific item*.

Coping behaviors differed by gender (Table 5). Women displayed higher scores for following strict protective measures (C1; *p* = 0.002), shouting and crying to relieve emotions (C9; *p* < 0.001), and the use of medical and homeopathic treatments (C11; *p* < 0.001). Men showed higher scores for tobacco or alcohol use (C12; *p* < 0.001).

Differences were found between age groups for coping behaviors. HCWs over 50 years old were less likely to avoid doing overtime [C7; *F*_(3, 364)_ = 3.861, *p* = 0.01] and avoid media news [C8; *F*_(3, 364)_ = 4.285, *p* = 0.005] compared to participants aged 20–30 years old (*p* = 0.009; *p* = 0.026).

### Comparison Between Frontline and Non-Frontline Staff

Supplementary Table 5 presents mean scores of frontline and non-frontline workers for the different items of the questionnaire. Concerning immediate reactions, staff working in COVID units (frontline workers) were unhappier about working overtime during the outbreak [Q3; *t*_(366)_ = 2.775, *p* = 0.006], felt angrier because the workload was greater and more dangerous than for other doctors who had not been exposed to COVID-19 [Q10; *t*_(366)_ = 2.513, *p* = 0.012], had a stronger feeling of no longer being able take care of their children and family and had no energy left [Q11; *t*_(366)_ = 2.336, *p* = 0.021] and felt less happy when they were with their children and family members [Q12; *t*_(366)_ = −2.499, *p* = 0.013].

Concerning stressors, frontline workers felt less stressed by the daily report of the number of new infected cases [S9; *t*_(366)_ = −2.302, *p* = 0.022], about developing symptoms of the disease [S12; *t*_(366)_ = −1.982, *p* = 0.049], and by the lack of means and protective clothing [S14; *t*_(366)_ = −2.307, *p* = 0.022]. However, frontline workers felt more stressed about seeing stress or fear from colleagues [S13; *t*_(366)_ = 2.038, *p* = 0.042] and wearing protective clothing for a long time [S15; *t*_(366)_ = 2.072, *p* = 0.039].

For protective factors, only practice putting on protective gear was scored higher by frontline workers [P6; *t*_(366)_ = 2.268, *p* = 0.024].

No difference was noted between the two groups for coping behaviors except for avoiding media news about COVID-19 and related fatalities [C8; *t*_(366)_ = 2.215, *p* = 0.028], which was higher in frontline workers.

## Discussion

In Tunisia, during the COVID-19 pandemic, HCWs faced a particularly stressful situation, with a high risk of contamination, inadequate access to PPE, and social isolation, with the consequent emergence of anxiety and depressive symptoms. Searching the epidemiological literature on disease outbreaks, we found a paucity of studies regarding psychiatric services in similar situations (5, 6). This study represents a necessary effort to address the needs of HCWs in an attempt to develop cognitive, emotional and interpersonal skills that promote adaptive responses and contribute to both organizational and personal resilience.

We found that HCWs felt nervous or frightened in 76.3% of cases, which has been supported by previous studies, although their extent differs (7, 8). After the Middle East Respiratory Syndrome (MERS) epidemic in Korea in 2015, healthcare workers caring for MERS patients experienced higher rates of psychological distress than their counterparts not involved in MERS-related tasks (9). Specifically, these HCWs showed increased rates of sleep problems, hyperarousal and avoidance. Liu et al. recently identified stress-related symptom rates of 73.4%, anxiety rates of 44.7%, depression rates of 50.7%, and insomnia rates of 36.1% among 1,563 medical staff as a result of this epidemic (10). A recent study in China found that approximately half of the surveyed health-care workers reported moderate to severe anxiety symptoms, and approximately one-third reported a moderate to severe psychological impact (11). With regard to the situation in Italy, the risks of acute stress disorder, burnout, and full psychiatric disorders are currently very high among HCWs (12). During the 2003 SARS outbreak in Canada, Maunder et al. found that a stress management model was useful in describing expected stress reactions and helping staff adapt rather than considering these reactions as pathological (13).

The most important factors that motivated HCWs were their social and moral responsibilities, similar to what has been reported in Hunan during the COVID-19 epidemic and in other studies (7, 8). A sense of purpose and altruism are key factors of resilience. However, anxiety, fear, and anger can easily overwhelm them during a major public health emergency. Hobfoll et al. identified self-efficacy, instillation of hope, and social connectedness as being among the crucial elements for promoting resilience in populations affected by mass trauma (14). According to Albot et al., self-efficacy is probably the most important skill for HCWs (15).

Our results show that the most stressed are medical residents, young HCWs and women. After the 2003 SARS outbreak, Tam et al. found similar results: worse psychological outcomes were seen in HCWs that were of a younger age, female, nursing professionals, and those with poorer physical health (16). Many studies reiterated these findings: staff who were women (17–19) younger (20–25) or parents of dependent children were more vulnerable to psychological distress (7–13). Several studies reported that women were more vulnerable to stress, probably owing to their heavier family responsibilities. Those who had children showed a higher risk of stress because of the fear of infecting their children and the family stigma of not being able to care for their children if they were contaminated (7, 16). Within several findings, nurses were generally more at risk for stress than doctors (26–28), apart from two studies that reported the opposite conclusions (29, 30).

In our study, nurses were not more nervous or frightened than doctors, perhaps because doctors had a deep understanding of the dangers of COVID-19, so they were more prone to anxiety and fear (28).

Staff who were older or who had greater clinical experience experienced less stress and less anxiety, which is in agreement with other findings (16, 20, 31, 32). The exceptions were in two studies of staff caring for patients with COVID-19, when older age was a risk factor for psychological symptoms (23, 33).

Keeping their families safe from infection was a main concern of HCWs. Goulia et al. reported similar results during the A/H1N1 influenza pandemic (26). Ensuring the care of HCW family members would enhance workforce confidence and availability, but the feasibility and advisability of family priority is yet to be determined (34).

Other major stressors were concerns of committing aseptic errors that could spread the disease, lack of PPE, and seeing their patients die or their colleagues contaminated. These findings have been reported in numerous studies, which have highlighted that access to adequate PPE and better control of disease transmission improved the psychological state of HCWs (8, 13, 16, 18, 35–37).

Another important stress factor is physical exhaustion. This involves arranging working time for caregivers with regular breaks. Some HCWs may need alternative housing to reduce the risk of contamination of family members (38). During these breaks, HCWs should be provided with food, with the opportunity to contact their families to alleviate concerns. During infectious disease epidemics, support from family and friends, as well as a positive attitude, has previously been shown to reduce stress (39). Similar results were also found in our study. Positive attitudes and discussions with family and friends were key elements of coping among HCWs.

In addition to these factors, we found that the ability to communicate with leaders reduces stress, which is consistent with the results of previous studies. Clear communication with regular and accurate updates on the epidemic should be provided to HCWs to address their uncertainty and fear (39). Tait Shanafelt et al. asked HCWs about the demands they made on their leaders during the COVID-19 pandemic and summarized them in 5 points: listen to me, protect me, prepare me, support me, and take care of me. HCWs want to be part of the development of plans and strategies to respond to the pandemic. A final request from health professionals—even if only implicitly acknowledged—is “honor me” (40). Honor is a powerful expression of gratitude and could be used to strengthen the compassion of HCWs who risk their lives on the job.

Another key component of a coping strategy is religious practice, which contributed to reduced stress for 87.2% of participants. This seems to be a specificity of our population. Shechter et al. found similar results: engaging with faith-based religion and/or spirituality (23%), yoga (25%), and/or meditation (23%) were major coping behaviors among HCWs (41). Albott et al. reported that spending time with religious, faith-based or spiritual practice is one of the factors of resilience (15). Learning mindfulness techniques for HCWs could also be of help (42).

Few HCWs (27.17%) expressed a desire to consult a psychiatrist or psychologist to reduce their stress, which is concordant with the findings of Chen Q. In China, medical staff are less likely to seek help from a psychologist or to express their emotions when compared with medical staff in Western countries. Many HCWs mentioned that they did not need a psychologist but needed more rest and enough PPE (6).

We found little to no differences between exposed and non-exposed health-care workers, confirming that, at the onset of the pandemic, the whole health-care system was impacted by the COVID-19 epidemic. Frontline workers felt more heavily the workload and consequences on their family life as in previous works (43). They felt less stressed about developing symptoms but had concerns about seeing stress or fear from colleagues and wearing protective clothing for a long time. Concerning coping behaviors, they avoided media news more. A study comparing healthcare workers with high- and low-risk exposure during the peak of the 2003 SARS outbreak, also found similarly elevated levels of perceived stress at the onset of the epidemic (44). However, in a 1-year follow-up, perceived stress decreased in the low-risk group, but increased in the high-risk group with significantly greater depression ratings at 1-year follow-up, which was partially mediated by stress related to contact with SARS (44). In the 1–2 years following the outbreak, frontline workers had higher levels of distress and PTSD. These results suggest that working in the front line is not an independent risk factor for worse mental health outcomes at the onset of the epidemic but can become one later in time.

The stress factors and the measures that can reduce stress were thus similar in Tunisia as in other countries affected by COVID-19. In addition to the coping behaviors found in other countries, some coping factors appeared to be specific to our population, such as the roles of family and friend support and religious and faith practice. Furthermore, we have evidenced little demand for psychological support, contrary to Western literature. Although in our context, mental illness is increasingly accepted and psychiatrists are increasingly consulted, HCWs wanted to seek the help of a psychologist or psychiatrist only in about one-third of the cases. For this reason, the psychological support offered by our institution has not been well-accepted by the HCWs.

### Limitations

This is a short-term (1 month) cross-sectional observational study. However, the psychological impact of COVID-19 may increase in the near future, implying the need for future studies.

In conclusion, major sources of psychological distress among HCWs are young age, female gender, lack of work experience, and fear for the safety of the family. Protective factors are availability of PPE, reassurance about family welfare, clear communication with leaders and access to reliable information. Coping behaviors adopted by HCWs are mainly based on family and friend exchanges and religious and spiritual practices rather than assistance from psychiatrists or psychologists. Learning mindfulness techniques for HCWs could also be of help.

An effective intervention for this case highlights a need to pay attention to a vulnerable group that includes the youngest and women. Identification of vulnerable individuals must be performed early for a better intervention, especially because stress could predispose them to depression and anxiety in the coming months (45). Results indicate that clear and empowering communication with leaders would be of great help to HCWs and that the mentoring of younger professionals by their elders is essential.

We believe that these results are important because, thanks to these findings, we have been able to establish recommendations and a strategy for improving the resilience of HCWs (see Psychosocial accompaniment and support in Supplementary Material). We hope that these findings will help decision-makers promote the will to protect HCWs in the current and future epidemics. As a hospital, patient care quality has to be at the center of its mission, and so, the safety and resilience of those delivering that care is intrinsically related to the level of quality. A burned out, stressed out, overworked, under-appreciated and anxious care giver ultimately will give compromised care.

This study has been of great help to us in dealing now with the second health crisis (second wave of this epidemic, which is more serious and more threatening). A long-term evaluation of this intervention is needed.

## Data Availability Statement

The original contributions presented in the study are included in the article/Supplementary Material, further inquiries can be directed to the corresponding author/s.

## Ethics Statement

The studies involving human participants were reviewed and approved by local committee for the protection of personnel of the military hospital of TUNIS CLPP. The patients/participants provided their written informed consent to participate in this study.

## Author Contributions

HeS, HE, and HG: study design, data collection, data analysis, statistical analyses, and manuscript writing. KT and NS: study design, statistical analyses, and manuscript writing. AB, WS, CB, and HiS: statistical analyses and manuscript writing. AO, KL, MH, and MF: manuscript writing. All authors contributed to the article and approved the submitted version.

## Conflict of Interest

The authors declare that the research was conducted in the absence of any commercial or financial relationships that could be construed as a potential conflict of interest.

## References

[1] National Observatory of New and Emerging Diseases. COVID-19 in Tunisia, Situation Update of May 28, 2020. (2020). Available online at: https://www.onmne.tn/wp-content/uploads/2020/10/COVID-19-dernier-bulletin-28052020.pdf (accessed May 29, 2020).

[2] Lee SH Juang YY Su YJ Lee HL Lin YH Chao CC. Facing SARS: psychological impacts on SARS team nurses and psychiatric services in a Taiwan general hospital. Gen Hosp Psychiatry. (2005) 27:352–8. 10.1016/j.genhosppsych.2005.04.007 16168796 PMC7132375

[3] Haozheng C Baoren T Jing M Limin C Lei F Yongfang J. Psychological impact and coping strategies of frontline medical staff in Hunan between January and March 2020 during the outbreak of coronavirus disease 2019 (COVID-19) in Hubei, China. Med Sci Monit. (2020) 26:e924171. 10.12659/MSM.924171 32291383 PMC7177038

[4] Roskam I Raes ME Mikolajczak M. Exhausted parents: development and preliminary validation of the parental burnout inventory. Front Psychol. (2017) 8:163. 10.3389/fpsyg.2017.00163 28232811 PMC5298986

[5] Lai J Ma S Wang Y Cai Z Hu J Wei N. Factors associated with mental health outcomes among health care workers exposed to coronavirus disease 2019. JAMA Netw Open. (2020) 3:e203976. 10.1001/jamanetworkopen.2020.3976 32202646 PMC7090843

[6] Chen Q Liang M Li Y Guo J Fei D Wang L. Mental health care for medical staff in China during the COVID-19 outbreak. Lancet Psychiatry. (2020) 7:e15–6. 10.1016/S2215-0366(20)30078-X 32085839 PMC7129426

[7] Koh D Lim MK Chia SE Ko SM Qian F Ng V. Risk perception and impact of severe acute respiratory syndrome (SARS) on work and personal lives of health care workers in Singapore: what can we learn? Med Care. (2005) 43:676–82. 10.1097/01.mlr.0000167181.36730.cc 15970782

[8] Khalid I Khalid TJ Qabajah MR Barnard AG Qushmaq IA. Healthcare workers emotions, perceived stressors and coping strategies during a MERS-CoV outbreak. Clin Med Res. (2016) 14:7–14. 10.3121/cmr.2016.1303 26847480 PMC4851451

[9] Lee SM Kang WS Cho AR Kim T Park JK. Psychological impact of the 2015 MERS outbreak on hospital workers and quarantined hemodialysis patients. Compr Psychiatry. (2018) 87:123–7. 10.1016/j.comppsych.2018.10.003 30343247 PMC7094631

[10] Liu S Yang L Zhang C Xiang YT Liu Z Hu S. Online mental health services in China during the COVID-19 outbreak. Lancet Psychiatry. (2020) 7:e17–8. 10.1016/S2215-0366(20)30077-8 32085841 PMC7129099

[11] Wang C Pan R Wan X Tan Y Xu L Ho CS. Immediate psychological responses and associated factors during the initial stage of the 2019 coronavirus disease (COVID-19) epidemic among the general population in China. Int J Environ Res Public Health. (2020) 17:1729. 10.3390/ijerph17051729 32155789 PMC7084952

[12] Sani G Janiri D Di Nicola M Janiri L Ferretti S Chieffo D. Mental health during and after the COVID-19 emergency in Italy. Psychiatry Clin Neurosci. (2020) 74:372. 10.1111/pcn.13004 32248608

[13] Maunder RG Lancee WJ Rourke S Hunter JJ Goldbloom D Balderson K. Factors associated with the psychological impact of severe acute respiratory syndrome on nurses and other hospital workers in Toronto. Psychosom Med. (2004) 66:938–42. 10.1097/01.psy.0000145673.84698.18 15564361

[14] Hobfoll SE Watson P Bell CC Bryant RA Brymer MJ Friedman MJ. Five essential elements of immediate and mid-term mass trauma intervention: empirical evidence. Psychiatry. (2007) 70:283–369. 10.1521/psyc.2007.70.4.283 18181708

[15] Albott CS Wozniak JR Mc Glinch BP Wall MH Gold BS Vinogradov S. Battle buddies: rapid deployment of a psychological resilience intervention for healthcare workers during the COVID-19 pandemic. Anesth Analg. (2020) 131:43–54. 10.1213/ANE.0000000000004912 32345861 PMC7199769

[16] Tam CW Pang EP Lam LC Chiu HF. Severe acute respiratory syndrome (SARS) in Hong Kong in 2003: stress and psychological impact among frontline healthcare workers. Psychol Med. (2004) 34:1197–204. 10.1017/S0033291704002247 15697046

[17] Tang L Pan L Yuan L Zha L. Prevalence and related factors of post-traumatic stress disorder among medical staff members exposed to H7N9 patients. Int J Nurs Sci. (2016) 4:63–7. 10.1016/j.ijnss.2016.12.002 31406720 PMC6626070

[18] Bukhari EE Temsah MH Aleyadhy AA Alrabiaa AA Alhboob AA Jamal AA. Middle East respiratory syndrome coronavirus (MERS-CoV) outbreak perceptions of risk and stress evaluation in nurses. J Infect Dev Ctries. (2016) 10:845–50. 10.3855/jidc.6925 27580330

[19] Zhu Z Xu S Wang H Liu Z Wu J Li G. COVID-19 in Wuhan: sociodemographic characteristics and hospital support measures associated with the immediate psychological impact on healthcare workers. EClinicalMedicine. (2020) 24:100443. 10.1016/j.eclinm.2020.100443 32766545 PMC7311903

[20] Chong MY Wang WC Hsieh WC Lee CY Chiu NM Yeh WC. Psychological impact of severe acute respiratory syndrome on health workers in a tertiary hospital. Br J Psychiatry. (2004) 185:127–33. 10.1192/bjp.185.2.127 15286063

[21] Austria-Corrales F Cruz-Valdés B Herrera-Kiengelher L Vázquez-García JC Salas-Hernández J. Burnout syndrome among medical residents during the influenza A H1N1 sanitary contigency in Mexico. Gac Med Mex. (2011) 147:97–103. 21527961

[22] Nickell LA Crighton EJ Tracy CS Al-Enazy H Bolaji Y Hanjrah S. Psychosocial effects of SARS on hospital staff: survey of a large tertiary care institution. CMAJ. (2004) 170:793–8. 10.1503/cmaj.1031077 14993174 PMC343853

[23] Wu P Fang Y Guan Z Fan B Kong J Yao Z. The psychological impact of the SARS epidemic on hospital employees in China: exposure, risk perception, and altruistic acceptance of risk. Can J Psychiatry. (2009) 54:302–11. 10.1177/070674370905400504 19497162 PMC3780353

[24] Sim K Chong PN Chan YH Soon WS. Severe acute respiratory syndrome related psychiatric and post traumatic morbidities and coping responses in medical staff within a primary health care setting in Singapore. J Clin Psychiatry. (2004) 65:1120–7. 10.4088/JCP.v65n0815 15323599

[25] Matsuishi K Kawazoe A Imai H Ito A Mouri K Kitamura N. Psychological impact of the pandemic (H1N1) 2009 on general hospital workers in Kobe. Psychiatry Clin Neurosci. (2012) 66:353–60. 10.1111/j.1440-1819.2012.02336.x 22624741

[26] Goulia P Mantas C Dimitroula D Mantis D Hyphantis T. General hospital staff worries, perceived sufficiency of information and associated psychological distress during the A/H1N1 influenza pandemic. BMC Infect Dis. (2010) 10:322. 10.1186/1471-2334-10-322 21062471 PMC2990753

[27] Wong TW Yau JK Chan CL Kwong RS Ho SM Lau CC. The psychological impact of severe acute respiratory syndrome outbreak on healthcare workers in emergency departments and how they cope. Eur J Emerg Med. (2005) 12:13–8. 10.1097/00063110-200502000-00005 15674079

[28] Poon E Liu KS Cheong DL Lee CK Yam LY Tang WN. Impact of severe respiratory syndrome on anxiety levels of front-line health care workers. Hong Kong Med J. (2004) 10:325–30. 15479961

[29] Chan AOM Huak CY. Psychological impact of the 2003 severe acute respiratory syndrome outbreak on health care workers in amedium size regional general hospital in Singapore. Occup Med. (2004) 54:190–6. 10.1093/occmed/kqh027 15133143 PMC7107861

[30] Lung FW Lu YC Chang YY Shu BC. Mental symptoms in different health professionals during the SARS attack: a follow-up study. Psychiatr Q. (2009) 80:107–16. 10.1007/s11126-009-9095-5 19247834

[31] Oh N Hong N Ryu DH Bae SG Kam S Kim KY. Exploring nursing intention, stress, and professionalism in response to infectious disease emergencies: the experience of local public hospital nurses during the 2015 MERS outbreak in South Korea. Asian Nurs Res. (2017) 11:230–6. 10.1016/j.anr.2017.08.005 28991605 PMC7104949

[32] Lancee WJ Maunder RG Goldbloom DS Coauthors for the Impact of SARS Study. Prevalence of psychiatric disorders among Toronto hospital workers one to two years after the SARS outbreak. Psychiatr Serv. (2008) 59:91–5. 10.1176/ps.2008.59.1.91 18182545 PMC2923654

[33] Xing J Sun N Xu J Geng S Li Y. Study of the mental health status of medical personnel dealing with new coronavirus pneumonia. PLoS ONE. (2020) 15:e0233145. 10.1371/journal.pone.0233145 32428041 PMC7237008

[34] Adams JG Walls RM. Supporting the health care workforce during the COVID-19 global epidemic. JAMA. (2020) 323:1439–40. 10.1001/jama.2020.3972 32163102

[35] Kim JS Choi JS. Factors influencing emergency nurse's burnout during an outbreak of middle east respiratory syndrome coronavirus in Korea. Asian Nurs Res. (2016) 10:295–9. 10.1016/j.anr.2016.10.002 PMC7104920 28057317

[36] Wong WC Lee A Tsang KK Wong SY. How did general practitioners protect themselves, their family, and staff during the SARS epidemic in Hong Kong? J Epidemiol Community Health. (2004) 58:180–5. 10.1136/jech.2003.015594 14966227 PMC1732708

[37] Chan SS Leung GM Tiwari AF Salili F Leung SS Wong DC. The impact of work-related risk on nurses during the SARS outbreak in Hong Kong. Fam Community Health. (2005) 28:274–87. 10.1097/00003727-200507000-00008 15958885

[38] Kisely S Warren N McMahon L Dalais C Henry I Siskind D. Occurrence, prevention, and management of the psychological effects of emerging virus outbreaks on health care workers: rapid review and meta-analysis. BMJ. (2020) 369:m1642. 10.1136/bmj.m1642 32371466 PMC7199468

[39] Xiang YT Yang Y Li W Zhang L Zhang Q Cheung T. Timely mental health care for the 2019 novel coronavirus outbreak is urgently needed. Lancet Psychiatry. (2020) 7:228–9. 10.1016/S2215-0366(20)30046-8 32032543 PMC7128153

[40] Shanafelt T Ripp J Trockel M. Understanding and addressing sources of anxiety among health care professionals during the COVID-19 pandemic. JAMA. (2020) 323:2133–4. 10.1001/jama.2020.5893 32259193

[41] Shechter A Diaz F Moise N Anstey DE Ye S Agarwal S. Psychological distress, coping behaviors, and preferences for support among New York healthcare workers during the COVID-19 pandemic. Gen Hosp Psychiatry. (2020) 66:1–8. 10.1016/j.genhosppsych.2020.06.007 32590254 PMC7297159

[42] Sidi H. The psychological sequelae during mental health and COVID-19 pandemic: learning from the past for today's coping styles. Med and Health. (2020) 15:1–4. 10.17576/MH.2020.1501.01

[43] Lu W Wang H Lin Y Li L. Psychological status of medical workforce during the COVID-19 pandemic: a cross-sectional study. Psychiatry Res. (2020) 288:112936. 10.1016/j.psychres.2020.112936 32276196 PMC7195354

[44] McAlonan GM Lee AM Cheung V Cheung C Tsang KW Sham PC. Immediate and sustained psychological impact of an emerging infectious disease outbreak on health care workers. Can J Psychiatry. (2007) 52:241–7. 10.1177/070674370705200406 17500305

[45] Woon LS Sidi H Nik-Jaafar NR Leong-Bin-Abdullah MFI. Mental health status of university healthcare workers during the COVID-19 pandemic: a post-movement lockdown assessment. Int J Environ Res Public Health. (2020) 17:9155. 10.3390/ijerph17249155 33302410 PMC7762588

