# Supplementary material for: Immediate Psychological Responses, Stress Factors, and Coping Behaviors in Military Health-Care Professionals During the COVID-19 Pandemic in Tunisia
Source: Front Psychiatry. 2021 May 20;12:622830. doi: 10.3389/fpsyt.2021.622830 (PMC8172775; doi:10.3389/fpsyt.2021.622830)
Supplement: Supplementary file 1 [file Data_Sheet_1.docx]

Supplemental Material

**Supplementary Table S1 : Medical staff characteristics by gender**

| **Characteristic** | **Male female P** |
| --- | --- |
| Mean age (years) ± SD | 36.7(11.84) 36.12(9.85) 0.62 |
| Married, N (%) | 128(57.4) 83(57.2) 0.11 |
| Having children, N (%) | 128(57.4) 82(56.5) 0.41 |
| Life habit, N (%)  Tobacco  Alcohol  Tobacco and alcohol  Nothing | 64(28.7) 5(3.4) < 0.001  15(6.7) 1(0.7)  19(8.5) -  125(56.1) 139(95.9) |
| Professional, N (%)  Nurse  Doctor  Medical resident  Medical Technician  Hospital staff | 107(48) 43(29.7) < 0.001  82(36.8) 56(38.6)  11(4.9) 23(15.9)  12(5.4) 14(9.7)  11(4.9) 9(9.2) |
| Workspace, N (%)  Military Hospital of Tunis  Medical Centers of the Military Units  Military Clinic  Specialized Military Medical Center    Military Hospital of Bizerte    Military Hospital of Gabes | 166(74.4) 113(77.9) 0.06  35(15.7) 13(9)  10(4.5) 11(7.6)  5(2.2) 7(4.8)  6(2.7) -  1(0.4) 1(0.7)  - - |
| Department, N (%)  Medicine department  Surgery department  Intensive care unit  Emergency department    Laboratory  Administration    Others | 89(39.9) 69(47.6 0.09  31(13.9) 27(18.6)  40(17.9) 11(7.6)  17(7.6) 12(8.3)  8(3.6) 6(4.1)  7(3.1) 6(4.1)  31(13.9) 14(9.7) |
| Work experience (years), mean (SD) | 11.89(10.09) 11.09(9.69) 0.21 |
| Work in COVID unit, N (%)  Yes  No | 67(30) 42(29) 0.82  156(70) 103(71) |

**Supplementary Table S2 : Medical staff characteristics in groups working or not in COVID unit**

| **Characteristic** | **Working in COVID unit**    **Yes No *P value*** |
| --- | --- |
| Mean age (years) ± SD | 35.53(10.43) 36.87(11.34) 0.29 |
| Sexe  Women  Men | 42(38.5) 103(39.8) 0.82  67(61.5) 156(57.9) |
| Married, N (%) | 62(56.9) 150(57.9) 0.9 |
| Having children, N (%) | 60(55) 82(56.55) 0.6 |
| Life habit, N (%)  Tobacco  Alcohol  Tobacco and alcohol  Nothing | 29(26.6) 12 (4.6) 0.01  4(3.7) 40(15.4)  9(8.3) 10(3.9)  67(61.5) 197(76.1) |
| Professional, N (%)  Nurse  Doctor  Medical resident  Medical Technician  Hospital staff | 52(47.7) 98(37.8) < 0.001  28(25.7) 110(42.5)  19(17.4) 15(5.8)  8(7.3) 18(6.9)  2(1.8) 18(6.9) |
| Workspace, N (%)  Military Hospital of Tunis  Medical Centers of the Military Units  Military Clinic  Specialized Military Medical Center    Military Hospital of Bizerte    Military Hospital of Gabes | 92(84.4) 187(72.2) 0.06  11(10.1) 37(14.3)  3(2.8) 18(6.9)  - 12(4.6)  2(0.9) 4(1.5)  1(0.9 1(0.4) |
| Department, N (%)  Medicine department  Surgery department  Intensive care unit  Emergency department    Laboratory  Administration    Others | 45(41.3) 113(43.6) 0.001  16(14.7) 42(16.2)  23(21.1) 28(10.8)  15(13.8) 14(5.4)  - 14(5.4)  1(0.9) 12(4.6)  9(8.3) 36(13.9) |
| Work experience (years), mean (SD) | 11.11(9.93) 11.77(9.94) 0.9 |

**Supplementary Table S3 Stressors by gender**

|  | Mean scores * | | *P*value |
| --- | --- | --- | --- |
|  | Women  (N= 145)  [mean (SD)] | Men  (N=223)  [mean (SD)] |  |
| S1-Infection of a co-worker | 2.32 (0.86) | 2.02 (1.00) | 0.003 |
| S2-Worried about infecting family | 2.72 (0.68) | 2.65 (0.74) | 0.391 |
| S3-Worried about getting infected | 2.13 (0.90) | 1.89 (0.97) | 0.016 |
| S4-The occurrence of errors at work that can lead to infections | 2.36 (0.86) | 2.26 (0.88) | 0.278 |
| S5-Watching infected patients die | 2.46 (0.80) | 2.11 (0.98) | 0.001 |
| S6-Not knowing when the outbreak will be contained | 1.62 (1.09) | 1.18 (1.08) | 0.000 |
| S7-Participating in the management of infected patients | 1.45 (1.08) | 1.03 (1.05) | 0.000 |
| S8-Lack of specific treatment for COVID | 2.32 (0.82) | 1.95 (1.00) | 0.000 |
| S9-The daily report of the number of new infected cases | 2.35 (0.76) | 2.06 (1.11) | 0.003 |
| S10-Feeling exhausted | 2.04 (0.93) | 1.55 (1.02) | 0.000 |
| S11-Observing symptoms of the disease in colleagues | 2.21 (0.92) | 1.87 (0.97) | 0.001 |
| S12-Developing symptoms of the disease | 2.20 (0.92) | 1.98 (0.99) | 0.028 |
| S13-Seeing stress or fear from colleagues | 2.13 (0.83) | 1.79 (0.98) | 0.001 |
| F14-Lack of means and protective clothing | 2.28 (0.99) | 2.21 (0.97) | 0.507 |
| S15-Wearing protective clothing for a long time | 1.77 (1.05) | 1.73 (0.99) | 0.690 |
| S16-Working outside of the team and the regular department | 1.71 (0.98) | 1.34 (1.05) | 0.001 |
| S17-Being repeatedly screened for infection | 1.35 (1.16) | 1.12 (1.04) | 0.046 |
| S18-Mandatory sanitary confinement | 1.65 (1.13) | 1.52 (1.11) | 0.297 |

Note: SD = Standard deviation

*mean of all responses to the item which was rated on a four-point scale (0= no distress; 1 = slightly; 2 = moderately; 3 = very much)

**Associations between highest stressors and Immediate reactions to the mission of health-care workers during COVID-19 outbreak**

We identified significant highest stressors using Student’s one-sample t-test with a test value equivalent to the mean of stressors (= 1.9). Significant stressors with a minimal Cohen’s d effect-size ≥ .02 were S1 (Infection of a co-worker; d = 0.254), S2 (Worried about infecting family; d = 1.087), S4 (The occurrence of errors at work that can lead to infections; d = 0.461), S5 (Watching infected patients die; d = 0.374), S8 (Lack of specific treatment for COVID-19; d = 0.208), S9 (The daily report of the number of new infected cases; d = 0.286) and S14 (Lack of means and protective clothing; d = 0.353) (all *p*s < 0.001). Supplementary Table S6 indicates Pearson correlations between highest stressors and Immediate reactions of health-care workers. To control for multiple comparisons, only significant correlations with a p-value ≤ 0.001 were considered for further analyses. When Immediate reactions were associated with multiple stressors, we conducted Stepwise linear regressions to identify the best predictor(s) for each Immediate reaction. The best predictors for Q2 (You have felt nervous or frightened in the ward) were S8 (p = 0.011) and S1(p = 0.014); S14 (p = 0.002) and S8 (p = 0.01) for Q3 (You were unhappy about working overtime during the outbreak); S4 (p = 0.006) and S9 (p = 0.023) for Q5 (You try to avoid COVID-19 positive patients or suspect of infection) ; S1 (p = 0.001) for Q6 (You try to avoid your colleagues working in COVID units); S9 (p < 0.001) for Q11 (When I come home, I feel unable to take care of my children or family members and have no energy) and S8 (p < 0.001) for Q14 (I feel that I take care of my children in a mechanical way and am not able to show them my affection). S2 was not significantly associated with an immediate reaction after correction for multiple comparisons.

**Supplementary Table S4 Associations between highest stressors and Immediate reactions to the mission of health-care workers during COVID-19 outbreak**

| Pearson Correlations  (*r-value)* |  |  |  |  |  |  |  |  | |  |  |  |  |  |  |
| --- | --- | --- | --- | --- | --- | --- | --- | --- | --- | --- | --- | --- | --- | --- | --- |
|  |  | Q1 | Q2 | Q3 | Q4 | Q5 | Q6 | Q7 | Q8 | Q9 | Q10 | Q11 | Q12 | Q13 | Q14 |
| S1 |  | 0.121 | 0.171 | 0.164 | 0.025 | 0.158 | 0.170 | 0.120 | 0.001 | 0.009 | 0.055 | 0.161 | -0.077 | 0.050 | 0.141 |
| *p*-value |  | 0.020 | 0.001 | 0.002 | 0.630 | 0.002 | 0.001 | 0.036 | 0.978 | 0.866 | 0.293 | 0.002 | 0.142 | 0.443 | 0.007 |
| S2 |  | 0.057 | 0.014 | 0.046 | 0.031 | 0.037 | 0.019 | -0.006 | -0.120 | -0.120 | 0.021 | 0.102 | -0.063 | 0.009 | 0.017 |
| *p*-value |  | 0.276 | 0.792 | 0.383 | 0.553 | 0.480 | 0.715 | 0.921 | 0.021 | 0.021 | 0.684 | 0.051 | 0.228 | 0.888 | 0.739 |
| S4 |  | 0.103 | 0.091 | 0.085 | 0.034 | 0.184 | 0.145 | 0.101 | 0.095 | 0.119 | 0.131 | 0.107 | 0.069 | 0.073 | 0.129 |
| *p*-value |  | 0.049 | 0.084 | 0.105 | 0.520 | < .001 | 0.005 | 0.079 | 0.070 | 0.023 | 0.012 | 0.041 | 0.190 | 0.266 | 0.014 |
| S5 |  | 0.096 | -0.022 | -0.056 | 0.038 | -0.021 | 0.002 | 0.040 | -0.113 | -0.074 | -0.035 | 0.086 | 0.047 | -0.083 | 0.100 |
| *p*-value |  | 0.093 | 0.700 | 0.333 | 0.505 | 0.709 | 0.966 | 0.509 | 0.049 | 0.195 | 0.546 | 0.134 | 0.415 | 0.232 | 0.080 |
| S8 |  | 0.107 | 0.174 | 0.167 | 0.033 | 0.110 | 0.110 | 0.107 | 0.067 | 0.078 | 0.096 | 0.171 | -0.011 | 0.173 | 0.166 |
| *p*-value |  | 0.040 | < .001 | 0.001 | 0.531 | 0.035 | 0.034 | 0.063 | 0.201 | 0.133 | 0.065 | 0.001 | 0.829 | 0.008 | 0.001 |
| S9 |  | 0.085 | 0.168 | 0.157 | 0.104 | 0.176 | 0.115 | 0.112 | 0.067 | 0.088 | 0.111 | 0.178 | 0.028 | 0.106 | 0.076 |
| *p*-value |  | 0.104 | 0.001 | 0.003 | 0.045 | < .001 | 0.028 | 0.051 | 0.203 | 0.093 | 0.033 | < .001 | 0.595 | 0.104 | 0.145 |
| S14 |  | 0.092 | 0.160 | 0.186 | 0.078 | 0.085 | 0.026 | 0.019 | 0.077 | 0.020 | 0.122 | 0.098 | 0.072 | 0.042 | 0.011 |
| *p*-value |  | 0.079 | 0.002 | < .001 | 0.136 | 0.103 | 0.626 | 0.748 | 0.141 | 0.708 | 0.019 | 0.061 | 0.170 | 0.519 | 0.832 |

**Supplementary Table S5: Comparison between frontline and non-frontline staff.**

|  |  | Working in COVID unit | |  | |
| --- | --- | --- | --- | --- | --- |
|  | YES | NO | |  | |
|  | Mean (SD) | Mean (SD) | | | P value |
| Q1 | 2.14±0.86 | | 2.11±0.90 | | 0.75 |
| Q2 | 1.12±0.83 | | 0.98±0.74 | | 0.11 |
| Q3 | 1.04±0.89 | | 0.77±0.78 | | 0.006 |
| Q4 | 1.13±1.06 | | 0.91±1.00 | | 0.06 |
| Q5 | 0.86±1.01 | | 1.08±1.09 | | 0.057 |
| Q6 | 0.72±0.96 | | 0.77±0.98 | | 0.66 |
| Q7 | 0.44±0.81 | | 0.45±0.86 | | 0.95 |
| Q8 | 0.13±0.44 | | 0.12±0.44 | | 0.78 |
| Q9 | 0.22±0.58 | | 0.13±0.42 | | 0.11 |
| Q10 | 0.86±0.89 | | 0.62±0.79 | | 0.01 |
| Q11 | 1.29±1.02 | | 1.02±0.94 | | 0.02 |
| Q12 | 1.62±1.06 | | 1.93±1.09 | | 0.01 |
| Q13 | 0.84±0.81 | | 0.66±0.88 | | 0.14 |
| Q14 | 1.05±0.91 | | 0.85±0.91 | | 0.058 |
| S1 | 2.22±0.91 | | 2.11±0.97 | | 0.31 |
| S2 | 2.77±0.64 | | 2.64±0.74 | | 0.11 |
| S3 | 1.94±0.98 | | 2.01±0.94 | | 0.55 |
| S4 | 2.23±0.92 | | 2.33±0.84 | | 0.35 |
| S5 | 2.18±0.97 | | 2.28±0.90 | | O.36 |
| S6 | 1.43±1.14 | | 1.32±1.09 | | 0.40 |
| S7 | 1.24±1.17 | | 1.17±1.04 | | 0.59 |
| S8 | 1.96±0.96 | | 2.15±0.94 | | 0.08 |
| S9 | 1.99±0.94 | | 2.22±0.86 | | 0.02 |
| S10 | 1.75±1.04 | | 1.74±1.01 | | 0.97 |
| S11 | 2.01±0.97 | | 2.00±0.96 | | 0.92 |
| S12 | 1.91±0.95 | | 2.13±0.98 | | 0.04 |
| S13 | 2.08±0.92 | | 1.86±0.94 | | 0.04 |
| S14 | 2.05±1.07 | | 2.32±0.92 | | 0.02 |
| S5 | 1.91±0.97 | | 1.68±1.02 | | 0.03 |
| S16 | 1.43±1.05 | | 1.51±1.03 | | 0.47 |
| S17 | 1.29±1.10 | | 1.18±1.09 | | 0.39 |
| S18 | 1.50±1.09 | | 1.61±1.14 | | 0.40 |
| C1 | 2.57±0.64 | | 2.46±0.70 | | 0.13 |
| C2 | 2.05±0.97 | | 2.07±0.90 | | 0.86 |
| C3 | 2.33±0.90 | | 2.46±0.83 | | 0.17 |
| C4 | 1.87±0.88 | | 1.99±0.90 | | 0.22 |
| C5 | 1.86±0.90 | | 2.01±0.87 | | 0.13 |
| C6 | 1.65±1.01 | | 1.61±0.93 | | 0.74 |
| C7 | 1.19±1.01 | | 1.16±0.97 | | 0.79 |
| C8 | 1.48±0.89 | | 1.25±1.00 | | 0.02 |
| C9 | 0.57±0.84 | | 0.43±0.74 | | 0.12 |
| C10 | 1.10±0.96 | | 1.00±0.88 | | 0.34 |
| C11 | 0.45±0.88 | | 0.45±0.74 | | 0.97 |
| C12 | 0.43±0.78 | | 0.435±0.7 | | 0.42 |
| C13 | 1.55±0.94 | | 1.54±0.97 | | 0.86 |
| C14 | 0.34±0.64 | | 0.35±0.64 | | 0.93 |
| P1 | 2.54±0.66 | | 2.41±0.80 | | 0.11 |
| P2 | 2.55±0.61 | | 2.45±0.77 | | 0.17 |
| P3 | 2.59±0.78 | | 2.63±0.66 | | 0.66 |
| P4 | 2.61±0.62 | | 2.59±0.68 | | 0.78 |
| P5 | 2.65±0.64 | | 2.68±0.54 | | 0.61 |
| P6 | 2.55±0.71 | | 2.35±0.79 | | 0.02 |
| P7 | 1.69±1.16 | | 1.91±1.06 | | 0.10 |
| P8 | 2.05±0.98 | | 2.15±0.92 | | 0.37 |
| P9 | 2.38±0.74 | | 2.32±0.85 | | 0.49 |
| P10 | 2.28±0.86 | | 2.34±0.86 | | 0.52 |
| P11 | 2.78±0.65 | | 2.88±2.42 | | 0.18 |
| P12 | 2.57±0.67 | | 2.59±0.66 | | 0.86 |
| P13 | 1.60±1.24 | | 1.71±1.22 | | 0.45 |
| P14 | 2.11±0.96 | | 2.18±0.93 | | 0.52 |
| P15 | 2.35±0.81 | | 2.21±0.90 | | 0.14 |
| P16 | 1.80±1.17 | | 1.79±1.04 | | 0.94 |

*Mean was rated on a four-point scale (0= not at all; 1 = slightly; 2 = moderately; 3 = very much)

**Psychosocial accompaniment and support:**

- Recognition of staff efforts

- Focusing on listening rather than on reassurance

- Peer encouragement, increased support from family and friends

- Organization of a periodical debriefing in the presence of the hospital director and the members of the steering committee to discuss the encountered difficulties, find solutions to remedy them and reinforce everyone in their professional value

- Respect of resting time

- Providing a place for relaxation, making contact with the family, doing sport (at least 15 minutes of physical exercise per day), for religious, confessional or spiritual practices, for reading, meditation

- Fostering positive emotions: expressing gratitude, feeling compassion; listening to music; spending time with friends, family, laughing

- On line training in stress management techniques (meditation, relaxation,)

- On line training in techniques for interviewing and managing an anxious or bereaved patient.

- Setting up a listening and psychological support unit available to the staff 24 hours a day.
